# Fetal Lead Exposure at Each Stage of Pregnancy as a Predictor of Infant Mental Development

**DOI:** 10.1289/ehp.9067

**Published:** 2006-07-19

**Authors:** Howard Hu, Martha María Téllez-Rojo, David Bellinger, Donald Smith, Adrienne S. Ettinger, Héctor Lamadrid-Figueroa, Joel Schwartz, Lourdes Schnaas, Adriana Mercado-García, Mauricio Hernández-Avila

**Affiliations:** 1 Department of Environmental Health, Harvard School of Public Health, Boston, Massachusetts, USA; 2 Channing Laboratory, Department of Medicine, Brigham and Women’s Hospital, Harvard Medical School, Boston, Massachusetts, USA; 3 Centro de Investigación en Salud Poblacional, Instituto Nacional de Salud Pública, Cuernavaca, Morelos, México; 4 Department of Neurology, Children’s Hospital, Harvard Medical School, Boston, Massachusetts, USA; 5 Department of Biology and Environmental Toxicology, University of California at Santa Cruz, Santa Cruz, California, USA; 6 Instituto Nacional de Perinatologia, Mexico City, Mexico

**Keywords:** bone, IQ, lead, plasma, pregnancy, neurodevelopment

## Abstract

**Background:**

The impact of prenatal lead exposure on neurodevelopment remains unclear in terms of consistency, the trimester of greatest vulnerability, and the best method for estimating fetal lead exposure.

**Objective:**

We studied prenatal lead exposure’s impact on neurodevelopment using repeated measures of fetal dose as reflected by maternal whole blood and plasma lead levels.

**Methods:**

We measured lead in maternal plasma and whole blood during each trimester in 146 pregnant women in Mexico City. We then measured umbilical cord blood lead at delivery and, when offspring were 12 and 24 months of age, measured blood lead and administered the Bayley Scales of Infant Development. We used multivariate regression, adjusting for covariates and 24-month blood lead, to compare the impacts of our pregnancy measures of fetal lead dose.

**Results:**

Maternal lead levels were moderately high with a first-trimester blood lead mean (± SD) value of 7.1 ± 5.1 μg/dL and 14% of values ≥10 μg/dL. Both maternal plasma and whole blood lead during the first trimester (but not in the second or third trimester) were significant predictors (*p* < 0.05) of poorer Mental Development Index (MDI) scores. In models combining all three trimester measures and using standardized coefficients, the effect of first-trimester maternal plasma lead was somewhat greater than the effect of first-trimester maternal whole blood lead and substantially greater than the effects of second- or third-trimester plasma lead, and values averaged over all three trimesters. A 1-SD change in first-trimester plasma lead was associated with a reduction in MDI score of 3.5 points. Postnatal blood lead levels in the offspring were less strongly correlated with MDI scores.

**Conclusions:**

Fetal lead exposure has an adverse effect on neurodevelopment, with an effect that may be most pronounced during the first trimester and best captured by measuring lead in either maternal plasma or whole blood.

The findings of a wide variety of international studies on the impacts of lead exposure on mental development persuaded many countries to progressively reduce the amount of lead exposure deemed safe during childhood. Since 1991, the U.S. Centers for Disease Control and Prevention (CDC) has recommended 10 μg/dL (0.48 μmol/L) as the pediatric blood lead screening action guideline (CDC 1991), with recent research (Canfield et al. 2003) and pooled analyses of seven prospective studies (Lanphear et al. 2005) prompting consideration of further reductions.

A related issue that has received less attention is the extent to which prenatal lead exposure may produce adverse outcomes. This issue has emerged as a potentially large public health problem because of two recent insights. First, substantial fetal lead exposure can occur from mobilization of maternal skeletal lead stores, which, in turn, can persist many years after external lead exposure has declined (Gulson et al. 2003; Hu and Hernandez-Avila 2002). Second is the growing appreciation of the fetal nervous system’s exquisite sensitivity to neurotoxins (Mendola et al. 2002).

Until now, few epidemiologic studies have used designs that allow the neurodevelopmental impacts of prenatal lead exposure to be distinguished from those of postnatal lead exposure. Among these, some have shown an inverse association between prenatal lead exposure and infant neurodevelopment (Bellinger et al. 1987; Dietrich et al. 1987; Ernhart et al. 1987; Shen et al. 1998) and some have not (Cooney et al. 1989; McMichael et al. 1988). Some found associations with neurodevelopment that attenuated over subsequent years (Bellinger et al. 1992; Dietrich et al. 1991; Ernhart et al. 1987), whereas others found relations that were stable over time (Wasserman et al. 1997, 2000).

An important factor that might contribute to inconsistency across studies is variability in the assessment and timing of dose to the fetus. Some studies measured maternal whole blood lead during the second and third trimesters and at delivery (Baghurst et al. 1987; Schnaas et al. 2006), whereas others took measures in the first or second trimester (Dietrich et al. 1987), in mid-pregnancy and at delivery (Wasserman et al. 1997), or at delivery only (Cooney et al. 1989; Ernhart et al. 1986). Some studies relied solely on umbilical cord blood lead level as the index of prenatal exposure (Bellinger et al. 1987). One study measured perinatal maternal bone lead level as an index of mobilizable maternal lead burden during the course of pregnancy (Gomaa et al. 2002).

The toxicokinetics of lead in the maternal–fetal unit are poorly understood. Lead levels in different compartments and at different stages of pregnancy are only modestly correlated, suggesting that each measure captures different aspects of fetal exposure (Baghurst et al. 1987). It is well known from the experimental literature that the vulnerability of developing organ systems, including the brain, to environmental toxicants can vary widely over the course of pregnancy (Mendola et al. 2002). Thus, it is plausible that lead exposure may be particularly neurotoxic during a specific trimester.

Recent evidence also suggests that whole blood lead levels in a pregnant woman might not be the optimal marker for lead concentrations in the fetal brain. Over 99% of lead in whole blood is bound to red cells and thus not available to cross the placenta (Goyer 1990); instead, it is the < 1% of lead in the plasma compartment of blood that is of greatest interest in terms of fetal exposure. Recent data suggest that there are significant interindividual differences in the ratio of red cell lead to plasma lead (Hu 1998; Lamadrid-Figueroa 2006), making maternal whole blood lead levels potentially unreliable as a proxy for plasma lead and fetal exposure (Chuang et al. 2001; Goyer 1990).

To date, no study of fetal lead neurotoxicity has included the biomarker measurements needed to compare whole blood and plasma lead levels during each trimester of pregnancy as predictors of infant neurodevelopment. It is such a comparison that we report here.

## Materials and Methods

### Study subjects

Subjects were recruited between May 1997 and July 1999 from 2,273 women approached during prenatal visits at one of three clinics of the Mexican Institute of Social Security (IMSS) in Mexico City. Women were eligible if they had a confirmed positive β-human chorionic gonadotropin test or were trying to become pregnant, lived in Mexico City, and were willing to participate in the 3-year follow-up study protocol. Of the 2,273 women approached, 1,502 (66%) declined to be enrolled. We applied the following exclusion criteria to the 771 (34%) women who were willing to participate (percent excluded in parentheses): having plans to leave the area in the following 5 years (3.7%); having a psychiatric disorder (0%); daily consumption of alcoholic beverages (0%); addiction to illegal drugs (0%); continuous use of prescription drugs (0%); diagnosis of high-risk pregnancy (10.9%), preeclampsia (0.9%), renal or circulatory disease including hypertension (8.4%), or gestational diabetes (0.7%); suffering from seizures that required medical treatment (0.3%); and being pregnant with > 14 weeks of gestation (15.3%). A total of 280 already pregnant women were recruited; 182 women with a negative pregnancy test declared an intention to become pregnant in the near future and were also recruited. Of the latter group, 47 became pregnant, agreed to participate, and were enrolled in the cohort comprising a total of 327 pregnant women.

Of these 327 women, 216 continued the full follow-up and bore children who were evaluated for the Bayley Mental Development Index (MDI) (Bayley 1993) at 24 months of age. Of these 216 mother–infant pairs, 146 met the following inclusion criteria: child born with at least 37 weeks of gestational age; at least one valid measurement of plasma lead during any of the three visits made during pregnancy; complete information on maternal age and IQ; and child’s blood lead level at 24 months of age, sex, weight, and height.

All mothers were informed about the study; those who agreed to participate read and signed a letter of informed consent. The research protocol was approved by the Ethics Committees of the National Institute of Public Health of Mexico, the Harvard School of Public Health, the Brigham and Women’s Hospital, the University of California, and the participating hospitals.

### Blood and plasma lead measurement in mothers

Blood and plasma samples were collected during each prenatal visit of the mothers to the Center for Environmental Health Research of the American British Cowdray (ABC) Hospital in Mexico City. Visits were scheduled at 12, 24, and 34 weeks of pregnancy, and samples were classified as corresponding to first, second, or third trimester according to the timing of these visits. Subjects were instructed to fast overnight before sample collection. Before venipuncture, each subject’s arm was washed with ultrapure water and disinfected with reagent-grade alcohol. Three milliliters of venous whole blood was collected with a butterfly catheter (19 gauge) into a low-lead container (Vacutainer B-D 367734; Becton-Dickinson, Franklin Lakes, NJ, USA) for blood lead analysis, and 13 cm^3^ venous blood was then collected into a polyethylene tube containing 100 ISP (international units) sodium heparin (H-3393; Sigma Chemical Company, St. Louis, MO, USA), processed, and shipped to the trace metal facility at the University of California, Santa Cruz, for measurement of whole blood lead and plasma lead using ultra-clean methods detailed elsewhere (Hernandez-Avila et al. 1998; Smith et al. 1998). All samples were analyzed using inductively coupled plasma mass spectrometry (ICP-MS; Thermo Finnigan, Bremen, Germany). Potential contamination by lead from hemolyzed red cells was assessed by measuring levels of plasma iron and free hemoglobin using sensitive methods previously described in detail (Smith et al. 1998). Accordingly, 18 samples were determined to be contaminated and excluded from further analyses.

### Children’s blood lead measurement

Umbilical cord and infant venous blood samples at 24 months were collected in trace metal–free tubes. Due to the logistical constraints posed by the collection of samples during birth from multiple hospitals and at unpredictable hours, we obtained data on cord blood on only 57% of the mothers participating in this study. Samples were analyzed for lead using an atomic absorption spectrometry (AAS) instrument (model 3000; PerkinElmer, Chelmsford, MA, USA) at the metals laboratory of the ABC Hospital, which participates in the external validation protocol of the Wisconsin Laboratory of Hygiene. The Pearson correlation coefficient between all available measurements by AAS and those by ICP-MS was 0.93 (in mothers). Precision was similar using either measuring technique; standard deviations were not significantly different (*p* = 0.32); and accuracy was comparable (with difference in means < 1.0 μg/dL).

### Measurement of child development and potential confounders

Infant development at 24 months was assessed by trained personnel using the Bayley Scales of Infant Development II–Spanish version (BSID-IIS) (Bayley 1993) using a standardized protocol described in a previous study by our research group (Gomaa et al. 2002). All assessors were blind to the children’s *in utero* and postnatal lead measurements. MDI scores at 24 months of age were considered the primary outcome. Information on demographic, socioeconomic, and other factors that could confound the relationship between lead and child development was collected. Maternal IQ was assessed using the Information, Comprehension, Similarities, and Block Design subtests of the Wechsler Adult Intelligence Score (Wechsler 1968).

### Statistical analysis

Descriptive statistics and appropriate transformations were performed before bivariate analyses. Outliers were identified using the ESD (Extreme Studentized Deviate) Many-Outlier procedure (Rosner 1983). We calculated Spearman correlation coefficients among the lead measurements. Height and weight data were transformed into *Z-*scores by using World Health Organization (WHO)/National Center for Health Statistics/ CDC reference data (WHO 1979) and interpreted as indices of a child’s nutritional status. Variables considered to be potential confounders based on biologic plausibility, regardless of statistical significance, and those significantly (*p* < 0.1) associated with MDI scores in bivariate analyses were included in multiple linear regression models; given these criteria, confounders included were child’s sex, blood lead at 24 months of age, height for age *z*-score and weight, as well as maternal age and intelligence quotient. All models featured log_e_-transformed lead measures because this procedure provided the best fit. We first generated “single-trimester” models, in which we evaluated the associations between MDI score and log_e_-transformed plasma and whole blood lead levels during each trimester of pregnancy adjusting for potential confounders. We generated “multitrimester” models, incorporating, in each model, the data from either plasma or whole blood lead concentrations from all three trimesters. We also ran models using maternal plasma lead or whole blood lead, averaged over all three trimesters.

To enable better comparability of the relative effects of plasma lead and blood lead, we compared effect estimates for a 1-SD change in each exposure metric. We carried out a similar analysis using log_e_-transformed cord blood lead levels as a proxy variable for prenatal lead exposure. To account for environmental exposure to lead in postnatal life, we also modeled MDI as a function of the child’s blood lead concentrations at 24 months of age.

**Figure 1:**
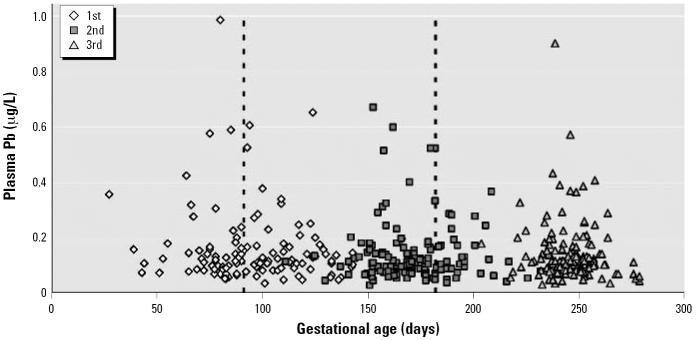
Plasma lead levels during pregnancy according to gestational age. Plasma lead smeasurements were taken at what were intended to be the first, second, and third trimesters. Dotted lines mark the 13th and 26th weeks of gestation.

Due to postponed visits to the research center visits of some women, misclassification of the timing of some of the visits occurred (e.g., a visit intended for the first trimester occurred during the second trimester; see Figure 1 for graphic display of misclassification). To account for the potential bias in results, we repeated the “intention to treat” analyses presented in this article, including only those observations that were correctly classified as corresponding to the first, second, and third trimesters of pregnancy according to actual gestational age.

Regression diagnostics were performed on all models to evaluate multicollinearity, distributional assumptions on the error term, and potentially influential data points. When the latter were detected, we fit new models excluding these observations. All statistical analyses were performed using STATA (STATA Statistical Software, release 8.0; StataCorp, College Station, TX, USA).

## Results

In the 146 mother–infant pairs in our final study group, no differences significantly greater than zero were noted in maternal age, number of years in school, IQ, and children’s hemoglobin, height, weight, and MDI when compared with mother–child pairs who participated but who did not meet the inclusion criteria for this analysis (*n* = 70; data not shown). Circulating levels of lead in the included mothers were moderately high, with mean (± SD) values for first-trimester whole blood lead of 70.7 ± 51.0 μg/L and 14% of values ≥ 10 μg/L (Table 1). (Whole blood lead values are expressed in this article as micrograms per liter for ease of comparison with plasma levels.) Both maternal plasma and whole blood lead followed a U-shaped pattern over the course of pregnancy, reaching their lowest points during the second trimester and rising during the third trimester.

As expected, measurements of lead biomarkers in the three stages of pregnancy were moderately well correlated (all *p* < 0.05); Spearman correlations between blood lead measurements at different stages of pregnancy (mean = 0.72; range, 0.67–0.81) were, on average, higher than their plasma lead counterparts (mean = 0.62; range, 0.55–0.69). Cord blood lead was most highly correlated with maternal whole blood lead measured during the third trimester of pregnancy (*r* = 0.5436, *p* < 0.001). Cord blood lead concentrations were 10.6 μg/L lower, on average, than maternal whole blood lead levels at delivery. Children’s whole blood lead levels at 12 and 24 months of age were correlated (*r* = 0.58, *p* < 0.01) and lower, on average, than their cord blood lead levels.

Single-trimester models of MDI scores (Table 2) suggested a negative relationship between circulating lead in each trimester of pregnancy and MDI scores at 24 months of age, adjusting for maternal age and IQ and child’s concurrent blood lead, sex, weight and height-for-age *Z*-score. MDI was most strongly associated with lead concentrations during the first trimester for both plasma (standardized coefficient, −4.13; *p* = 0.03) and whole blood lead (standardized coefficient, −3.77; *p* = 0.04). Both maternal plasma and whole blood lead averaged over all three trimesters had associations with MDI of borderline significance (standardized coefficients of −3.52, *p* = 0.07; and −3.11, *p =* −0.10, respectively).

When we repeated the analysis using only those measurements correctly classified in each trimester of pregnancy, we found that lead concentrations during the first trimester were significantly associated with a decrease in MDI at 24 months of age. The estimated coefficients in the first trimester (*n* = 56) were −6.39 (*p* = 0.04) and −6.94 (*p* = 0.04) points per log micrograms per liter of plasma and whole blood lead, respectively. The coefficients for the second trimester plasma and whole blood lead levels (*n* = 102) were much smaller (−1.73, *p* = 0.38; and −3.66, *p* = 0.16, respectively). Umbilical cord lead at birth and infant whole blood lead at 12 and 24 months were inversely but weakly (*p* > 0.20) associated with MDI at 24 months.

In multitrimester models (Table 3), the plasma lead model predicts that an increase of 1SD in log_e_-transformed plasma lead in the first trimester is associated with a 3.5-point lower MDI score at 24 months of age (*p* = 0.03). The corresponding increase in whole blood lead during the first trimester is associated with a 2.4-point lower MDI score at 24 months of age (*p* = 0.19). When first-trimester plasma lead concentrations were included in the model, plasma lead concentrations in the second and third trimester were not significantly associated with MDI. When both plasma and whole blood lead are simultaneously evaluated, although none of the standardized coefficients are statistically significant, the plasma lead coefficient is more than twice as great as its blood lead counterpart (−1.68, *p* = 0.36; and −0.77, *p* = 0.68, respectively).

**Figure 2:**
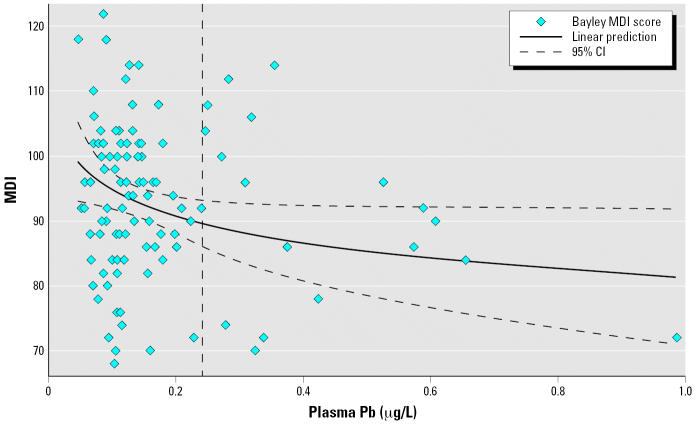
Plasma lead levels in the first trimester of pregnancy versus MDI scores at 24 months of age. CI, confidence interval. Curve indicates the best-fit model for the association between plasma lead levels and MDI scores, adjusting for plasma lead levels in the second and third trimesters, mother’s age and IQ, child’s blood lead levels at 24 months of age, sex and height-for-age *Z*-score. Vertical line marks average plasma lead concentration when whole blood lead equals 100 μg/L.

The logarithmic nature of the relationship between first-trimester plasma lead levels and MDI at 24 months of age is depicted in Figure 2. The vertical line represents the mean plasma lead (0.24 μg/L) corresponding to a whole blood lead concentration of 10 μg/L. The slope is steeper at lower levels. Linear regression models of the association using nontransformed plasma lead had a similar pattern. When the model is restricted to plasma lead observations below the median (0.1226 μg/L), an increase of 0.1 μg/L in plasma lead in the first trimester is associated with a 15-point decline in MDI score at 24 months of age, in contrast to a 4-point decline per 0.1-μg/L increase in plasma lead for observations above the median plasma lead—confirming that the nonlinear pattern is not an artifact of our transformation of the variable.

## Discussion

This study is the first of which we are aware that attempted to compare the relative influence on neurodevelopmental toxicity of two different biomarkers of fetal lead exposure at each stage of pregnancy. We found that both maternal blood lead and maternal plasma lead vary considerably over pregnancy; first-trimester levels of either measures were better than second- or third-trimester levels or levels averaged over all three trimesters at predicting infant neurobehavioral performance at age 24 months; and first-trimester maternal plasma lead levels were somewhat better than first-trimester maternal whole blood lead levels at predicting infant neurobehavioral performance at 24 months of age.

Our study had several limitations. Our sample size was modest, a reflection of the labor- and cost-intensive nature of collecting plasma samples using a rigorous protocol. Nevertheless, we were able to successfully distinguish and compare the relative contributions to neurodevelopment of trimester- and biomarker-specific measures of exposure. Our subjects were a small subset of women who had been initially approached in the clinics (*n* = 2,273), raising the issue of the generalizability of our study. However, the women included in our final sample did not differ significantly from other eligible subjects on key covariates, suggesting that our sample was quite representative of the women serviced by our participating clinics. Some of our observations were misclassified with respect to trimester; however, our results were very similar in the reanalysis using classification corrections. Indeed, the association between first-trimester lead exposure and infant neurodevelopment appeared to be even greater, a finding that suggests that there was downward bias due to improper assignment of second-trimester women to the first trimester category. We did not control for a summary measure of home conditions, such as the Home Observation for Measurement of the Environment (HOME) score; however, the absence of this covariate is unlikely to explain differences in effects among the three trimesters of lead exposure. Finally, offspring blood lead levels at 24 months did not significantly predict lower MDI score; on the other hand, our sample size, again, was modest, and in a separate analysis of the larger group of mother–infant pairs participating in this research (*n* = 294) that was not confined to women who had plasma lead measurements, we found a significant adverse impact of offspring blood lead levels at 24 months of age on 24-month MDI score (Tellez-Rojo et al. 2006).

The best-fitting model relating first-trimester plasma lead to 24-month MDI scores was one in which lead level was expressed as the natural logarithm of the measured value. This suggests that the shape of the dose–effect relationship is supralinear, with a steeper slope at lower plasma lead levels. This is consistent with the blood lead–IQ relationships in children reported by Canfield et al. (2003), in reanalyses of the Boston prospective study of children (Bellinger and Needleman 2003), and in pooled analyses that included several additional prospective studies (Lanphear et al. 2005). In quantitative terms, however, the rates of change over both ranges of plasma lead level were approximately twice as great as those reported by Canfield et al. (2003). Meta-analyses of multiple studies have converged on an estimate of a 2–3 IQ point decrement for each 10-μg/dL increase in postnatal blood lead level (International Program on Chemical Safety 1995; Pocock et al. 1994; Schwartz 1994), but these estimates might reflect mostly the region of the dose–effect relationship in which the slope is shallower. Moreover, blood lead is a surrogate measured with error for toxicologically available lead, and the larger effect size estimates for plasma lead suggest that many previous studies may have had effect estimates downwardly biased by measurement error.

We are not aware of previous studies for comparison that have included maternal measures of circulating lead at each stage of pregnancy. Although Schnaas et al. (2006) also studied lead exposure and neurobehavior in a cohort of Mexico City children from the *in utero* period to childhood (and found a significant adverse impact of *in utero* lead exposure), their observations began after the 12th week of pregnancy and thus precluded examination of the direct effects of first-trimester lead exposure.

In experimental studies, lead is known to affect a wide range of processes critical to central nervous system development, including differentiation (Alfano and Petit 1982; Petit and LeBoutillier 1979; Petit et al. 1983), myelination (Mendola et al. 2002), and synaptogenesis (Johnston and Goldstein 1998). Of these, differentiation is primarily a first-trimester event, making a targeting of this process as a possible explanation for our finding of a first-trimester dominant effect.

Mobilization of maternal bone lead stores has been clearly identified as a major source of fetal lead exposure (Gulson et al. 2003; Hu and Hernandez-Avila 2002), and elevated maternal bone lead stores can be expected in women with ongoing environmental or occupational exposures and in women who have retained bone lead burdens from earlier lead exposures. The women in our study fell into the latter category, having lived in Mexico City, where leaded gasoline was combusted until 1997. Some have suggested that fetal lead exposure resulting from the mobilization of maternal bone lead stores during pregnancy can be reduced by calcium supplementation (Gulson et al. 2004; Janakiraman et al. 2003). Our study suggests that if such a strategy were to prove useful in reducing lead exposure to the fetus, it would have to be implemented very early in pregnancy to maximize the benefit to fetal neurodevelopment.

Our findings do not mean that measurement of maternal plasma lead is likely to become a clinically useful environmental health tool. The methods required to measure plasma lead are laborious and require special and expensive equipment. However, this biomarker is a useful research tool in efforts to understand and detect the health impacts of environmental lead exposure.

In conclusion, we found that first-trimester measures of fetal lead exposure—particularly levels of lead in maternal plasma, but also levels of lead in maternal whole blood—were predictive of adverse neurodevelopment later in life, with an effect that was independent from that of postnatal lead exposure and that was stronger than the effects associated with second- or third-trimester measures. This is of major potential public health concern because lead remains a widespread environmental health hazard and current efforts at primary prevention have focused almost entirely on childhood rather than fetal exposure. If future research confirms this finding, ascertaining women at risk and identifying effective strategies for prevention of fetal lead exposure may become an important public health priority; moreover, it may be necessary to consider prepregnancy interventions, because our research suggests that screening and intervention any later than the first trimester may be too late to prevent the greatest fetal neurotoxic effects.

## Figures and Tables

**Table 1:** Characteristics of the study population of mother–infant pairs.

Characteristics	No.	Mean ± SD	Range
Mothers			
Age (years)	146	27.1 ± 5.3	15–43
IQ	146	89.1 ± 12.9	55–120
Whole blood lead (μg/L)			
First trimester	119	70.7 ± 51.0	14.9–435.9
Second trimester	136	60.8 ± 31.5	15.8–224.4
Third trimester	132	68.6 ± 42.3	15.3–330.8
Delivery	111	72.6 ± 43.3	15–324
Plasma lead (μg/L)			
First trimester	119	0.16 ± 0.14	0.04–0.99
Second trimester	136	0.14 ± 0.11	0.03–0.67
Third trimester	132	0.16 ± 0.24	0.03–2.63
Children			
Birth weight (g)	146	3,144 ± 359	2,125–4,000
Male sex (%)	76	52.05	
Blood lead (μg/L)			
Cord	83	62.0 ± 38.8	9–200
12 months	125	52.2 ± 34.1	9–204
24 months	146	47.9 ± 37.1	8–368
Height at 24 months (cm)	146	86.1 ± 3.0	74–93
Weight at 24 months (kg)	146	11.98 ± 1.55	9.4–19.3
Hemoglobin (g/dL)	135	12.4 ± 1.2	7.1–14.8
MDI score (at 24 months)	146	91.5 ± 11.6	68–122

**Table 2:** Single-trimester multivariate linear regression models for MDI of offspring (at 24 months of age) comparing markers of lead exposure at different times for blood lead and plasma lead.

Variable	No.	β	*p*-Value	95% CI
Pregnancy				
Blood lead (μg/L)				
First trimester	119	−4.13	0.04	−8.10 to −0.17
Second trimester	136	−4.08	0.06	−8.29 to 0.12
Third trimester	132	−2.42	0.23	−6.38 to 1.54
Average^a^	146	−3.52	0.10	−7.66 to 0.63
Plasma lead (μg/L)				
First trimester	119	−3.77	0.03	−7.12 to −0.42
Second trimester	136	−2.48	0.13	−5.74 to 0.77
Third trimester	132	−0.32	0.83	−3.38 to 2.74
Average^a^	146	−3.11	0.07	−6.53 to 0.31
Delivery				
Cord blood lead (μg/L)	83	−0.35	0.88	−4.72 to 4.03
Postnatal				
Child blood lead (μg/L)				
12 months	125	−2.38	0.23	−6.24 to 1.49
24 months	146	−1.00	0.50	−3.93 to 1.94

CI, confidence interval. Each model is adjusted for infant’s concurrent blood lead (24 months of age), sex, maternal age, current weight, height-for-age *Z*-score, and maternal IQ. Logarithmically transformed lead concentrations were used. Each line in the table represents a different model.

a The arithmetic mean of log-blood lead or log-plasma lead using all available measurements.

**Table 3:** Multivariate models of MDI of offspring (at 24 months of age) using either whole blood or plasma lead concentrations as markers of prenatal lead exposure at different trimesters of pregnancy.

	Plasma model (*R*^2^ = 0.22)		Blood model (*R*^2^ = 0.21)	
Variable	β	*p*-Value	β	*p*-Value
Pb first trimester^a^	−3.54	0.03	−2.40	0.19
Pb second trimester^a^	0.80	0.65	−1.29	0.56
Pb third trimester^a^	1.18	0.44	1.42	0.46
Current blood lead^b^	−0.01	0.62	−0.01	0.80
Sex^c^	3.64	0.13	3.50	0.15
Height-for-age *Z*-score	2.87	0.05	2.71	0.06
Current weight (kg)	−1.70	0.06	−2.00	0.02
Mother’s IQ	0.08	0.40	0.08	0.39
Mother’s age (years)	0.59	0.01	0.62	0.01
Intercept	84.25	< 0.01	87.18	< 0.01

These are the results of two multivariate regression models with either plasma lead or whole blood concentrations in different trimesters of pregnancy simultaneously included in each of the two models. Coefficients are mean change in MDI per increase of 1 SD in log_e_ lead concentrations, which allows for direct comparisons between the beta-coefficients of plasma lead versus blood lead.

a Plasma lead concentration (μg/L) in corresponding trimester.

b Infant whole blood lead (μg/L) at 24 months of age.

c Infant sex: 1 = male, 2 = female.
